# The Role of Architectural and Learning Constraints in Neural Network Models: A Case Study on Visual Space Coding

**DOI:** 10.3389/fncom.2017.00013

**Published:** 2017-03-21

**Authors:** Alberto Testolin, Michele De Filippo De Grazia, Marco Zorzi

**Affiliations:** ^1^Department of General Psychology and Padova Neuroscience Center, University of PadovaPadova, Italy; ^2^San Camillo Hospital IRCCSVenice, Italy

**Keywords:** connectionist modeling, unsupervised deep learning, restricted Boltzmann machines, autoencoders, sparseness, space coding, gain modulation, sensorimotor transformations

## Abstract

The recent “deep learning revolution” in artificial neural networks had strong impact and widespread deployment for engineering applications, but the use of deep learning for neurocomputational modeling has been so far limited. In this article we argue that unsupervised deep learning represents an important step forward for improving neurocomputational models of perception and cognition, because it emphasizes the role of generative learning as opposed to discriminative (supervised) learning. As a case study, we present a series of simulations investigating the emergence of neural coding of visual space for sensorimotor transformations. We compare different network architectures commonly used as building blocks for unsupervised deep learning by systematically testing the type of receptive fields and gain modulation developed by the hidden neurons. In particular, we compare Restricted Boltzmann Machines (RBMs), which are stochastic, generative networks with bidirectional connections trained using contrastive divergence, with autoencoders, which are deterministic networks trained using error backpropagation. For both learning architectures we also explore the role of sparse coding, which has been identified as a fundamental principle of neural computation. The unsupervised models are then compared with supervised, feed-forward networks that learn an explicit mapping between different spatial reference frames. Our simulations show that both architectural and learning constraints strongly influenced the emergent coding of visual space in terms of distribution of tuning functions at the level of single neurons. Unsupervised models, and particularly RBMs, were found to more closely adhere to neurophysiological data from single-cell recordings in the primate parietal cortex. These results provide new insights into how basic properties of artificial neural networks might be relevant for modeling neural information processing in biological systems.

## Introduction

Artificial neural network models aim at explaining human cognition and behavior in terms of the emergent consequences of a large number of simple, subcognitive processes (McClelland et al., 2010). Within this framework, the pattern seen in overt behavior (macroscopic dynamics of the system) reflects the coordinated operations of simple biophysical mechanisms (microscopic dynamics of the system), such as the propagation of activation and inhibition among elementary processing units. Though this general tenet is shared by all connectionist models, there is large variability in processing architectures and learning algorithms, which turns into varying degrees of psychological and biological realism (e.g., Thorpe and Imbert, 1989; O'Reilly, 1998). When the aim is to investigate high-level cognitive functions, simplification is essential (McClelland, 2009) and the underlying processing mechanisms do not need to faithfully implement the neuronal circuits supposed to carry out such functions in the brain. However, modelers should strive to consider biological plausibility if this can bridge different levels of description (Testolin and Zorzi, 2016).

Recent theoretical and technical progress in artificial neural networks has significantly expanded the range of tasks that can be solved by machine intelligence. In particular, the advent of powerful parallel computing architectures based on Graphic Processing Units (GPUs), coupled with the availability of “big data,” has allowed to create and train large-scale, hierarchical neural networks known as *deep neural networks* (LeCun et al., 2015, for review). These powerful learning systems achieve impressive performance in many challenging cognitive tasks, such as visual object recognition (Krizhevsky et al., 2012), speech processing (Mohamed et al., 2012) and natural language understanding (Collobert et al., 2011). However, while the impact of deep learning for engineering applications is undisputed, its relevance for modeling neural information processing in biological systems still needs to be fully evaluated (for seminal attempts, see Stoianov and Zorzi, 2012; Khaligh-Razavi and Kriegeskorte, 2014; Güçlü and van Gerven, 2015).

One critical aspect of most deep learning systems is the reliance on a feed-forward architecture trained with error backpropagation (Rumelhart et al., 1986), which has been repeatedly shown to yield state-of-the-art performance in a variety of problems (LeCun et al., 2015). However, the assumptions that learning is largely discriminative (e.g., classification or function learning) and that an external teaching signal is always available at each learning event (i.e., all training data is “labeled”) are clearly implausible from both a cognitive and a biological perspective (Zorzi et al., 2013; Cox and Dean, 2014). Reinforcement learning is a valuable alternative and it has already shown promising results when combined with deep learning (Mnih et al., 2015; Silver et al., 2016), but there is a broad range of situations where learning seems to be fully unsupervised and its only objective is that of discovering the latent structure of the input data in order to build rich, internal representations of the environment (Hinton and Sejnowski, 1999). We argue that more realistic neurocognitive models should therefore also exploit unsupervised forms of deep learning, where the objective is not to explicitly classify the input patterns but rather to discover internal representations by fitting a hierarchical generative model to the sensory data (Hinton, 2007, 2013; Zorzi et al., 2013). Compared to its supervised counterpart, this modeling approach emphasizes the role of feedback, recurrent connections (Sillito et al., 2006), which carry top-down expectations that are gradually adjusted to better reflect the observed data (Hinton and Ghahramani, 1997; Friston, 2010) and which can be used to implement concurrent probabilistic inference along the whole cortical hierarchy (Lee and Mumford, 2003; Gilbert and Sigman, 2007). Notably, top-down processing is also relevant for understanding attentional mechanisms in terms of modulation of neural information processing (Kastner and Ungerleider, 2000).

A powerful class of stochastic neural networks that learn a generative model of the data is that of Restricted Boltzmann Machines (RBMs), which can efficiently discover internal representations (i.e., latent features) using Hebbian-like learning mechanisms (Hinton, 2002). RBMs constitute the building block of hierarchical generative models such as Deep Belief Networks (Hinton and Salakhutdinov, 2006) and Deep Boltzmann Machines (Salakhutdinov, 2015). These unsupervised deep learning models have been successfully used to simulate a variety of cognitive functions, such as numerosity perception (Stoianov and Zorzi, 2012), letter perception (Testolin et al., under review), location-invariant visual word recognition (Di Bono and Zorzi, 2013), and visual hallucinations in psychiatric syndromes (Reichert et al., 2013). A similar approach has been used to simulate how early visual cortical representations are adapted to statistical regularities in natural images, in order to predict single voxel responses to natural images and identify images from stimulus-evoked multiple voxel responses (Güçlü and van Gerven, 2014). A temporal extension of RBMs has also been recently used to model sequential orthographic processing and spontaneous pseudoword generation (Testolin et al., 2016).

Unsupervised deep learning can be implemented using an alternative architecture based on autoencoders (Bengio et al., 2007), which are deterministic, feed-forward networks whose learning goal is to accurately reconstruct the input data into a separate layer of output units. Single-layer autoencoders are trained using error backpropagation, and can be stacked in order to build more complex, multi-layer architectures. However, despite the common view that RBMs and autoencoders could be considered equivalent (Ranzato et al., 2007), we note that their underlying architectural and learning assumptions are significantly different. In this study we empirically compare RBMs and autoencoders in terms of the type of internal encoding emerging in the hidden neurons. Moreover, we investigate how additional learning constraints, such as sparsity and limitation of computational resources (i.e., hidden layer size), could influence the representations developed by the networks. As a case study, we focus on the problem of learning visuospatial coding for sensorimotor transformations, which is a prominent example of how the emergentist approach based on learning in artificial neural networks has offered important insights into the computations performed by biological neurons (Zipser and Andersen, 1988).

Sensorimotor transformations refer to the process by which sensory stimuli are converted into motor commands. For example, reaching requires to map visual information, represented in retinal coordinates, into a system of coordinates that is centered on the effector. Coordinate transformations can be accomplished by combining sensory information with extra-retinal information, such as postural signals representing the position of eyes, head, or hand, thereby obtaining abstract representations of the space interposed between the sensory input and the motor output (Pouget and Snyder, 2000). Single-neuron recordings from monkey posterior parietal cortex have shown that the response amplitude of many neurons indeed depends on the position of the eyes, thereby unveiling a fundamental coding principle used to perform this type of signal integration (Andersen et al., 1985). The term *gain field* was coined to describe this gaze-dependent response of parietal neurons, and since then the notion of *gain modulation* has been generalized to indicate the multiplicative control of one neuron's responses by the responses of another set of neurons (Salinas and Thier, 2000). Another fundamental property unveiled by neuronal recordings is that the encoding of space used for coordinate transformations involves a variety of different, complementary frames of reference. For example, although many parietal neurons are centered on retinal coordinates (Andersen et al., 1985; Duhamel et al., 1992), others represent space using body-centered (Snyder et al., 1998) or effector-centered (Sakata et al., 1995) coordinate systems. Moreover, some neurons exhibit multiple gain modulation (Chang et al., 2009), suggesting more complex forms of spatial coding. For example, postural information related to both eye and head positions can be combined in order to encode “gaze” direction (Brotchie et al., 1995; Stricanne et al., 1996; Duhamel et al., 1997).

From a computational perspective, the seminal work of Zipser and Andersen (1988) showed that gain modulation could spontaneously emerge in supervised, feed-forward neural networks trained to explicitly map visual targets into head-centered coordinates, giving as input any arbitrary pair of eye and retinal positions. Similar results have been observed using more biologically-plausible learning settings, such as reinforcement learning (Mazzoni et al., 1991) and predictive coding (De Meyer and Spratling, 2011). Note that these learning settings assume that gain modulation emerges because the task implies to establish a mapping between different reference frames. However, it is unclear whether the form of modulation and the distribution of neuronal tuning functions is influenced by the type of learning algorithm and/or by the nature of the learning task (i.e., learning input-output mappings vs. unsupervised learning of internal representations). We also note that a popular alternative framework for modeling sensorimotor transformations is not based on learning, but rather stipulates that parietal neurons represent a set of basis functions that combine visual and postural information (for review, see Pouget and Snyder, 2000).

In summary, space coding represents an interesting case study for testing the adequacy of different neural network architectures and learning algorithms, because it provides a wealth of neurophysiological data (both at the population and single-neuron levels), and it departs from the classic problem of visual object recognition investigated in the large majority of deep learning research.

## Materials and methods

In this section we describe the space coding tasks used in our simulations, including training and test stimuli, the different learning architectures, and the procedures for analyzing the emergent neural representations.

### Space coding tasks

In this study we consider a visual signal in retinotopic coordinates and two different postural signals, one for eye position and another for a generic “effector,” which might represent, for example, the position of the hand. We do not consider the integration between different modalities (see Xing and Andersen, 2000, for a computational investigation of multimodal integration in several coordinate frames). We implemented three types of space coding tasks to test the different learning architectures.

#### Unsupervised learning with no coordinate transformation

**Figure 1:**
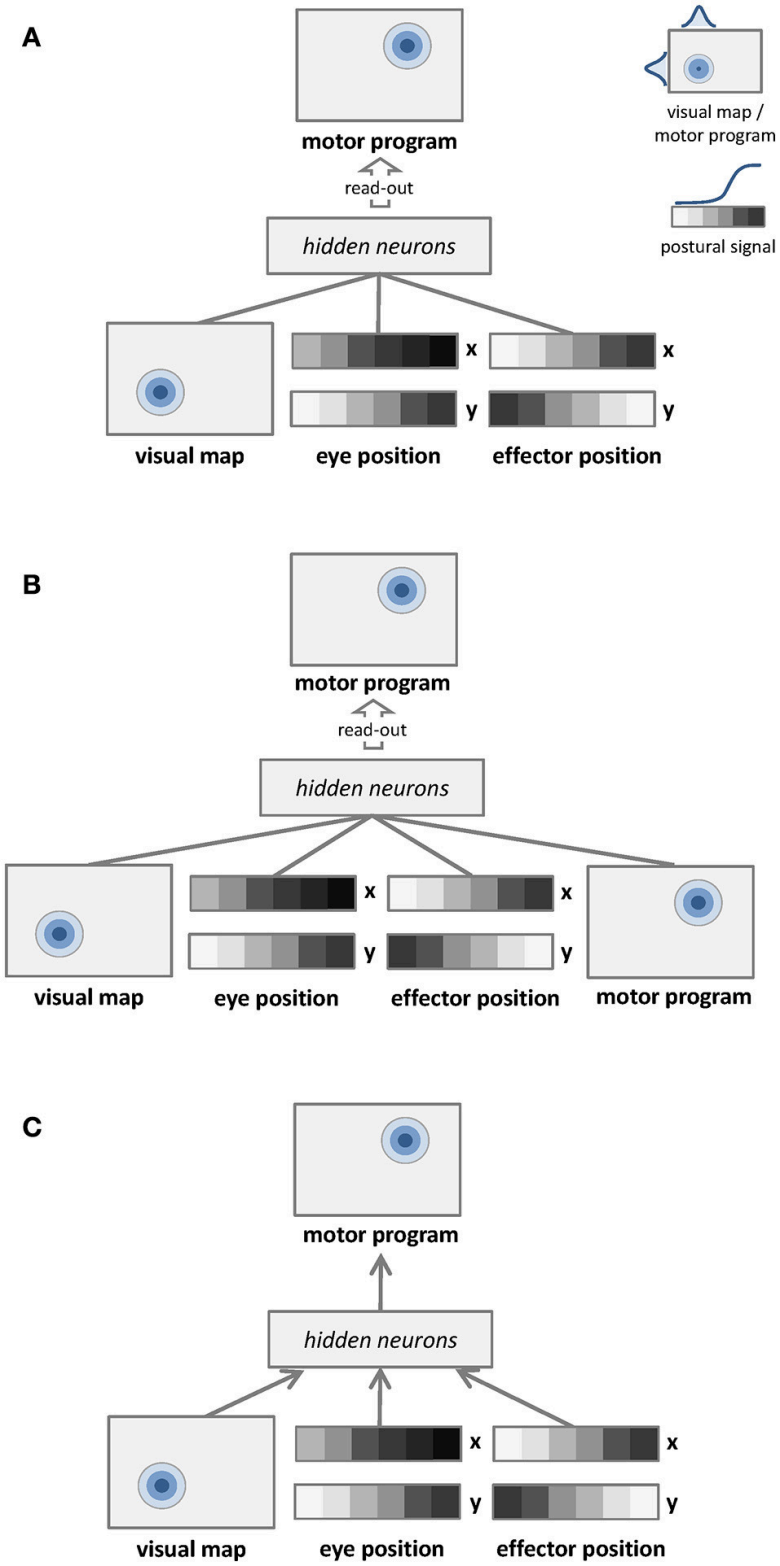
**Graphical representations of the learning architectures used to simulate the space coding tasks**. Undirected edges entail bidirectional (recurrent) connections, while directed arrows represent feed-forward connections. **(A)** Unsupervised learning with no coordinate transformation. **(B)** Unsupervised learning with coordinate transformation. **(C)** Supervised learning with coordinate transformation.

The first learning architecture is depicted in Figure 1A. Unsupervised learning is represented by undirected arrows, which connect the sensory input to a separate layer of hidden neurons. The input signal to the network consists of a visual map, which represents target location in retinotopic coordinates, and two postural maps, which represent eye and effector positions. The learning goal is only to build a compact representation of these input signals in the hidden layer, which is later read-out by a simple linear associator in order to establish a mapping with the corresponding motor program. Details of input and output representations are provided in Section Dataset and Stimuli. The unsupervised learning phase does not involve any coordinate transformation because information about the motor program is not available.

#### Unsupervised learning with coordinate transformation

The second learning architecture is depicted in Figure 1B. The input signal to the network still consists of a visual map and two postural maps, but in this case we also provide as input the corresponding motor program. In this setting the unsupervised learning phase implicitly involves coordinate transformation (i.e., different coordinate systems become associated). In order to compare the mapping accuracy of different learning architectures using the same method, the motor program is still read-out from hidden neurons via a simple linear associator.

#### Supervised learning with coordinate transformation

The third learning architecture is depicted in Figure 1C, and it corresponds to the model used by Zipser and Andersen (1988). The input is the same of the unsupervised architecture shown in Figure 1A, but in this case supervised learning (directed arrows) is used to establish an explicit mapping between input signals and motor programs. As for the previous architectures, accuracy of the motor program is also tested by read-out from hidden neurons via linear association.

### Dataset and stimuli

The representation format adopted for the sensory stimuli was the same used in previous computational investigations (Zipser and Andersen, 1988; Pouget and Snyder, 2000; De Filippo De Grazia et al., 2012), which is broadly consistent with neurophysiological data recorded in animals performing tasks involving coordinate transformations (e.g., Andersen et al., 1985).

The visual input to the models consisted in a real-valued vector representing the position of the stimulus as a Gaussian peak of activity in a specific location. These visible neurons simulate the activity of the cortical areas supplying retinotopic sensory information to the posterior parietal cortex. The retinotopic map consisted in a square matrix of 17 × 17 neurons, which employed a population code with Gaussian tuning functions (standard deviation = 4°). Visual receptive fields were uniformly spread between −9° and +9° with increments of 3°, both in the horizontal and vertical dimensions.

Four postural maps, each one consisting of 17 neurons, were used to represent the horizontal and vertical positions of the eye and the effector. These visible neurons used a sigmoid activation function (steepness parameter = 0.125) to represent postural information between −18 and +18°, with steps of 3°.

The motor program consisted in a real-valued vector representing the target position of the stimulus. Similarly to the retinotopic map, it was coded as a square matrix of 25 × 25 neurons, which employed a population code with Gaussian tuning functions to represent target position in coordinates centered on the effector (standard deviation = 6°). Motor programs were uniformly spread between −9° and +9° with increments of 3°, both in the horizontal and vertical dimensions.

In order to create the stimuli dataset, all possible combinations of visual input and postural signals were first generated, and the corresponding motor program (target location) was computed. We then balanced the patterns to ensure that target locations were equally distributed across the motor map to avoid position biases when decoding the motor program. This resulted in a total of 28,880 patterns, which were randomly split into a training set (20,000 patterns) and an independent test set (8,880 patterns). The latter was used to assess the generalization performance of the models.

### Learning architectures

Despite they differ in several aspects, Boltzmann machines and autoencoders can both be defined within the mathematical framework of energy-based models (Ranzato et al., 2007), where the learning objective is to carve the surface of an energy function so as to minimize the energies of training points and maximize the energies of unobserved points. A set of latent variables is used to learn an internal code that can efficiently represent the observed data points, and since the number of latent variables is usually smaller than that of the observed variables the encoding process can be interpreted as a form of dimensionality reduction (Hinton and Salakhutdinov, 2006). In this unsupervised setting, the model learns the statistical structure of the data without the need for any explicit, external label.

#### Restricted boltzmann machines (RBMs)

**Figure 2:**
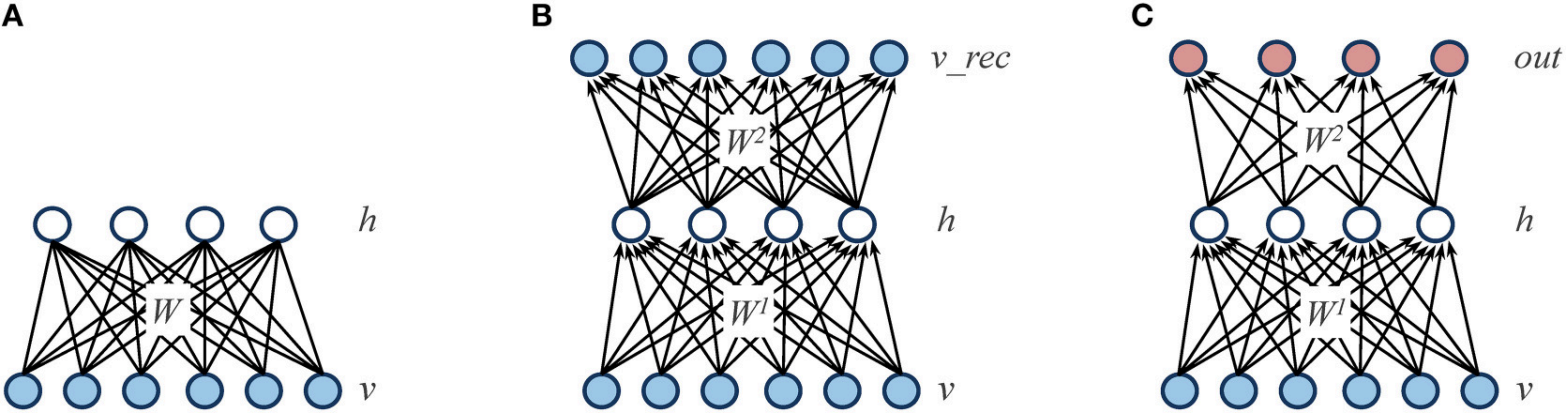
**Graphical representations of the different learning architectures used in the simulations. (A)** Restricted Boltzmann Machine (RBM): the learning objective is to accurately reconstruct the input patterns presented through the visible layer (*v*) by relying on a set of hidden units (*h*), which represent the latent structure of the data. The reconstruction is performed by using a weight matrix (*W*) that contains symmetric (i.e., undirected) connections. **(B)** Autoencoder: as for RBMs, the learning objective is to accurately reconstruct the input patterns presented through the visible layer (*v*) by relying on a set of hidden units (*h*). However, the reconstruction is performed on a separate layer of units (*v_rec*) by using two weight matrices (*W*^1^ and *W*^2^) that contain directed connections. **(C)** Feed-forward, supervised network: in contrast to RBMs and autoencoders, the learning objective is to minimize the mapping error between the input patterns presented through the visible layer (*v*) and a distinct set of output patterns presented through a dedicated layer (*out*).

Boltzmann machines are stochastic neural networks that use a set of hidden neurons to model the latent causes of the observed data vectors, which are presented to the network through a set of visible neurons (Ackley et al., 1985). In the “restricted” case, the network connectivity is constrained in order to obtain a bipartite graph (i.e., there are no connections within the same layer; see Figure 2A for a graphical representation). The behavior of the network is driven by an energy function *E*, which defines the joint distribution of the hidden and visible neurons by assigning a probability value to each of their possible configurations:

$$p(v,h)=\frac{e^{-E(v,\;h)}}{Z}$$

where *v* and *h* are the column vectors containing the values of visible and hidden neurons, respectively, and *Z* is the partition function. The energy function is defined as a linear combination of visible and hidden neurons' activation:

$$E(v,h)=-b^{T}v-c^{T}h-h^{T}Wv$$

where *W* is the matrix of connections weights, *b* and *c* are two additional parameters known as unit biases and *T* denotes the transpose operator. Since there are no connections within the same layer, hidden neurons are conditionally independent given the state of visible neurons (and vice versa). In particular, the activation probability of the neurons in each layer conditioned on the activation of the neurons in the opposite layer can be efficiently computed in one parallel step:

$$\begin{array}{lll} P(h_{j}=1|v) & = & \sigma (c_{j}+{\sum^{\text{​}}}_{i}w_{ij}v_{i})\\ P(v_{i}=1|h) & = & \sigma (b_{i}+{\sum^{\text{​}}}_{j}w_{ij}h_{j}) \end{array}$$

where σ is the sigmoid function, *c*_*j*_ and *b*_*i*_ are the biases of hidden and visible neurons (*h*_*j*_ and *v*_*i*_ respectively), and *w*_*ij*_ is the connection weight between *h*_*j*_ and *v*_*i*_. Learning in RBMs can be performed through maximum-likelihood, where each weight should be changed at each step according to a Hebbian-like learning rule:

$$\Delta W=\eta (v^{+}h^{+}-v^{-}h^{-})$$

where η represents the learning rate, *v*^+^*h*^+^ are the visible-hidden correlations computed on the training data (positive phase), and *v*^−^*h*^−^ are the visible-hidden correlations computed according to the model's expectations (negative phase). Model's expectations have been traditionally computed by running Gibbs sampling algorithms until the network reached equilibrium (Ackley et al., 1985). However, more efficient algorithms such as contrastive divergence (Hinton, 2002) speed-up learning by approximating the log-probability gradient. The reader is referred to Hinton (2010) and Zorzi et al. (2013) for more details about RBMs and for the discussion of hyper-parameters of the learning algorithm.

In our simulations, RBMs were trained using 1-step contrastive divergence with a learning rate of 0.03, a weight decay of 0.0002 and a momentum coefficient of 0.9, which was initialized to 0.5 for the first few epochs. Learning was performed using a mini-batch scheme, with a mini-batch size of 4 patterns, for a total of 100 learning epochs (reconstruction error always converged). Sparse representations were encouraged by forcing the network's internal representations to rely on a limited number of active hidden units, that is, by driving the probability *q* of a unit to be active to a certain desired (low) probability *p* (Lee et al., 2008). For logistic units, this can be practically implemented by first calculating the quantity *q-p*, which is then multiplied by a scaling factor and added to the biases of each hidden units at every weight update. When the sparsity constraint was applied, we always verified that the average activation of hidden units was indeed maintained below the desired level. All the simulations were performed using an efficient implementation of RBMs on graphic processors (Testolin et al., 2013). The complete source code is available for download^1^.

#### Autoencoders

Similarly to RBMs, autoencoders rely on a single layer of nonlinear hidden units to compactly represent the statistical regularities of the training data. However, autoencoders are feed-forward, deterministic networks trained with error backpropagation (Bengio et al., 2007). The training data is presented to a layer of input units, and the learning goal is to accurately reconstruct such input vector into a separate, output layer. An autoencoder is therefore composed of a set of encoding weights *W*^1^ that are used to compute the activation of hidden *h* units given the activation of input units *v*, and a set of decoding weights *W*^2^ that are used to compute the network reconstructions *v_rec* from the activations of hidden units:

$$\begin{array}{l} \;h=\sigma (W^{1}v+c)\\ v\_rec=\sigma (W^{2}h+b) \end{array}$$

where *b* and *c* are the vectors of output and hidden unit biases, and σ is the sigmoid function (see Figure 2B for a graphical representation). The error function *E* to be minimized corresponds to the average reconstruction error, which is quantified by the sum across all output units of the squared difference between the original and the reconstructed values:

$$E=\frac{1}{N}\overset{N}{\underset{n\;=1}{\sum^{\text{​}}}}\overset{K}{\underset{k\;=\;1}{\sum^{\text{​}}}}{\left(v_{k}-v\_rec_{k}\right)}^{2}+\beta^{*}\Omega_{sparsity}$$

where *K* is the number of output units and *N* is the number of training patterns. Similarly to RBMs, sparse representations can be induced by adding to the cost function a regularization term Ω_*sparsity*_ that takes a large value when the average activation value *q* of each hidden neuron diverges from a certain desired (low) value *p*. In particular, the sparsity constraint was implemented as the Kullback-Leibler divergence from *q* to *p*:

$$\begin{array}{l} \Omega_{sparsity}=\sum_{i=1}^{H}KL\left(p\;||\;q_{i}\right) \end{array}$$

where *H* is the number of hidden units. As for RBMs, when sparsity was applied we always verified that the average activation of hidden units was indeed maintained below the desired level.

In our simulations, we used an efficient implementation of autoencoders provided by the MATLAB Neural Network toolbox (Demuth and Beale, 1993). Learning was performed using standard scaled conjugate gradient descent (Møller, 1993) with adaptive learning rate, using a weight decay factor of 0.0002 and a batch processing scheme, for a total of 150 learning epochs (reconstruction error always converged).

#### Feed-forward, supervised networks

In order to better assess the impact of the learning regimen, we compared the unsupervised learning architectures described above with a standard, supervised architecture implemented as a feed-forward network with one hidden layer (Zipser and Andersen, 1988). Similarly to autoencoders, learning can be performed using error backpropagation (see Figure 2C for a graphical representation). We used an efficient implementation of feed-forward networks provided by the MATLAB Neural Network toolbox^2^. Learning rate was set to 0.05 and training was performed for a total of 2500 learning epochs (output error always converged).

### Testing procedure

For each experimental setting, we run 10 different networks in order to collect simulation statistics. In the results, we therefore always report mean values along with standard deviations.

#### Decoding internal representations by linear read-out

Following unsupervised learning, a linear read-out was performed from the internal (hidden layer) distributed representations of the networks in order to assess how well they could support a supervised mapping to the target motor program through a simple linear projection (Pouget and Snyder, 2000). The read-out was implemented using a linear neural network trained with the delta rule (Widrow and Hoff, 1960). Learning was performed for 250 epochs using mini-batches of 20 patterns. Learning rate was set to 0.07, and weight decay of 0.000001 was used as a regularizer. Classifier performance was always measured on the separate test set. Test errors always matched those obtained on the training set, indicating that the read-out was robust to overfitting.

The output of the classifier was first compared with the target motor program by computing the Root Mean Squared Error (RMSE) between the two matrices. However, a more useful performance measure was obtained by first decoding the Center Of Mass (COM) of the output distribution, which was then compared with the actual coordinates of the motor program. This measure allows to quantify the read-out error in degrees: following Zipser and Andersen (1988), the mapping was considered to be successful if the error was below the distance between the centers of the Gaussian tuning functions in the retinotopic map (i.e., 3°). If the latter mapping accuracy was not achieved, we did not consider the network for subsequent analyses. We found the RMSE and COM measures to be always consistent with each other, so we only report COM results.

#### Measuring single-neuron and population sparseness

An index of *single-neuron sparseness* was computed using a well-established procedure employed in neurophysiological investigations (Rolls and Tovee, 1995; Vinje and Gallant, 2000), which describes the activity fraction *a* of each neuron across stimuli as:

$$\begin{array}{l} a=\frac{{\left(\sum^{\text{​}}r_{i}/n\right)}^{2}}{\sum^{\text{​}}({r_{i}}^{2}/n)} \end{array}$$

where *r*_*i*_ is the firing rate of the neuron to the *i*-th stimulus in the set of *n* stimuli. This is a useful measure of the extent of the tail of the distribution, in this case of the firing rates of the neuron to each stimulus. Mean single-neuron sparseness for each network was then calculated by averaging the activity fraction *a* across all hidden neurons. A low value (minimum value is 0, maximum value is 1) indicates that the distribution has a long tail, which means that, on average, each neuron has high activation levels only for a small subset of input patterns. This method for quantifying sparseness has a number of advantages (Rolls and Tovee, 1995): (a) it results from formal analyses of the capacity of neural networks using an approach derived from theoretical physics (Treves and Rolls, 1991); (b) it can be applied both to binary neurons and to neurons with continuous (graded) firing rates; (c) it makes no assumption about the form of the firing rate distribution and (d) it makes no assumption about the mean and the variance of the firing rate.

Following Froudarakis et al. (2014) we also computed an index of *population sparseness*, on which the activity fraction is computed over the entire hidden layer, that is, by considering *r*_*i*_ as the firing rate of the *i*-th neuron and *n* as the total number of neurons. Mean population sparseness for each network was then calculated by averaging the activity fraction *a* across all stimuli. A low value of population sparseness indicates that, on average, each stimulus elicits high activations only for a small subset of hidden neurons.

#### Receptive fields emerging in the hidden neurons

In order to qualitatively assess the type of visual features extracted by individual hidden neurons, we first analyzed the weight matrices by separately plotting the strengths of the connections between each hidden neuron and all the visible neurons corresponding to the retinal input. Weights were plotted on a gray scale, with dark colors indicating strong inhibitory connections and light colors representing positive, excitatory connections. This allowed to assess whether hidden neurons learned location-specific receptive fields, for example by developing stronger projections to specific regions of the visual field.

#### Gain modulation indexes

We then analyzed the response of hidden neurons using a standard approach adopted in neurophysiological studies to assess gain modulation in parietal neurons (Andersen et al., 1985). First, we probed the hidden neurons in order to only select the “visual” ones, that is, those responding to the portion of input vectors representing the retinotopic map (De Filippo De Grazia et al., 2012). To this aim, we first recorded all hidden neurons' activations when the network received as input only all possible combinations of eye and effector positions (i.e., the retinotopic map and, if present, the motor program, were set to zero), and for each neuron we selected the positions corresponding to maximum activation. We then probed again each neuron, this time providing as input all possible retinotopic signals along with the preferred combination of postural signals. The neuron was considered as visual if its maximum activity differed by more than 10% from that recorded in the absence of visual input. Non-visual neurons were discarded from subsequent analyses^3^. We then computed a gain modulation index (GMI) for each neuron by recording its response to each target location as a function of eye and effector position (Pouget and Snyder, 2000). We first identified the combination of postural and retinal input producing the maximum neuron activation value. Starting from this input combination, we systematically varied each postural variable (one at a time, keeping all the others fixed) and computed gain modulation as the normalized ratio between the maximum and minimum activation values. Therefore, each neuron was characterized by four different GMIs, representing the gain for each postural variable with respect to horizontal and vertical axes. We finally sorted all hidden neurons into four different categories based on the combination of GMI indexes (using a threshold of 0.5 to establish modulation): (i) no modulation (i.e., purely visual neurons), (ii) modulation by eye position only, (iii) modulation by effector position only, and (iv) modulation by both eye and effector position.

## Results

Learning always converged for all models. For unsupervised models, convergence was monitored by measuring the mean reconstruction error on the whole training set. Autoencoders required more learning epochs to converge, but also achieved a lower reconstruction error compared to RBMs. This is probably due to the fact that autoencoders are natively real-valued. Existing real-valued extensions of RBMs (Cho et al., 2011) assume that the input values are normally distributed, which was not our case, so we preferred to use standard RBMs. Learning in the feed-forward, supervised models required almost 20 times more epochs to converge (the number of epochs required by each learning architecture is reported in Table 1).

**Table 1:** **Read-out errors for each learning architecture and space coding task, as a function of hidden layer size**.

**Space coding task**	**Layer size**	**RBMs**		**Autoencoders**		**Supervised Feed-forward**	
		**Read-out**	**Epochs**	**Read-out**	**Epochs**	**Read-out**	**Epochs**
No transformation	200	1.59 (0.08)	100	1.05 (0.05)	150		
	300	1.39 (0.07)	100	0.91 (0.04)	150		
	400	1.30 (0.08)	100	0.86 (0.04)	150		
	500	1.25 (0.04)	100	0.89 (0.02)	150		
	600	1.23 (0.05)	100	0.90 (0.02)	150		
	700	1.33 (0.04)	100	0.90 (0.03)	150		
Coordinate transformation	500	1.55 (0.15)	100	1.45 (0.05)	150	1.46 (0.06)	2,500
	600	1.47 (0.12)	100	1.46 (0.06)	150	1.45 (0.02)	2,500
	700	1.52 (0.11)	100	1.45 (0.05)	150	1.46 (0.05)	2,500
	800	1.57 (0.11)	100	1.47 (0.08)	150	1.47 (0.08)	2,500
	900	1.56 (0.16)	100	1.45 (0.07)	150	1.47 (0.04)	2,500

*Read-out errors are in degrees, and standard deviations are reported in parentheses. The “Epochs” column shows the number of epochs required by each learning architecture to converge*.

**Figure 3:**
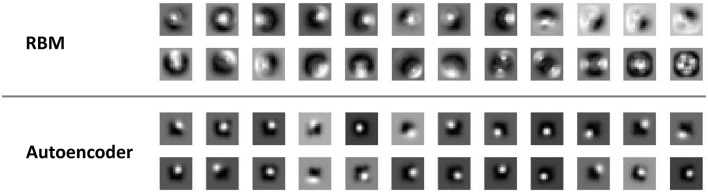
**Visual receptive fields**. Samples of receptive fields emerging from RBMs (top panel) and autoencoders (bottom panel) on the unsupervised learning task that did not require coordinate transformations. Similar receptive fields emerged from the unsupervised learning task involving coordinate transformations.

A first, qualitative analysis shows that RBMs and autoencoders developed different types of receptive fields. As shown in Figure 3, autoencoders learned homogeneous, location-specific receptive fields that uniformly covered the central regions of the visual input. On the other hand, while some neurons in the RBMs learned location-specific receptive fields resembling those of autoencoders, other neurons developed more complex receptive fields covering larger regions of the visual fields, sometimes also simultaneously covering symmetrical portions of the input image.

**Figure 4:**
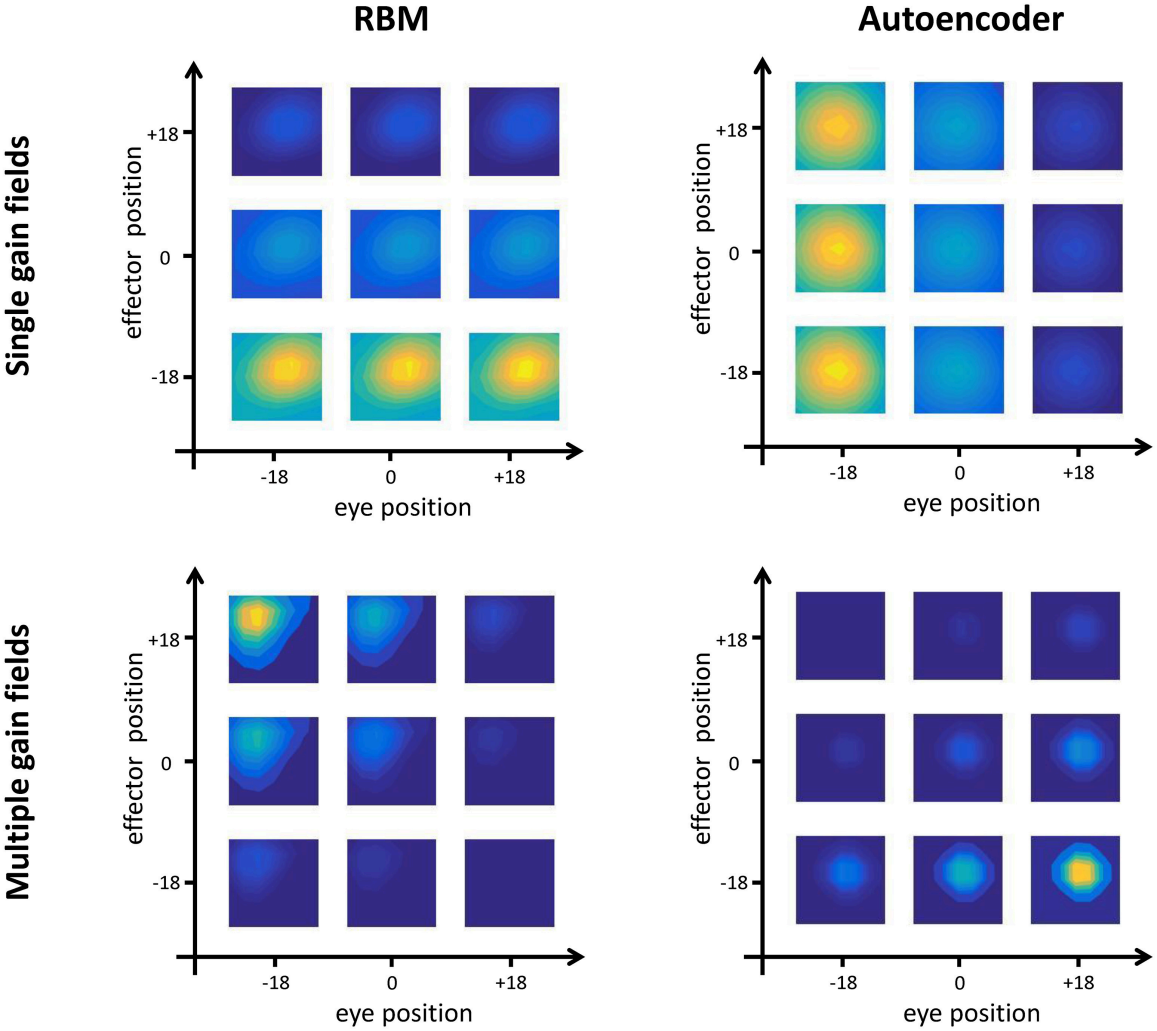
**Gain field coding**. Examples of single (top panels) and multiple (bottom panels) gain fields emerging in the hidden neurons of RBMs **(left)** and autoencoders **(right)**. Colors represent the amount of activation, with yellow indicating highest activation and dark blue indicating lowest activation. Single gain fields are characterized by a modulation of the neuron's activation that depends only on one postural signal (in the figure, effector position for the RBM and eye position for the autoencoder). In multiple gain fields, the activation is modulated by both signals.

The quantitative analyses (see Section Testing Procedure) allowed to group hidden neurons into different categories according to their response profiles. In line with empirical findings (Duhamel et al., 1997), there were always some neurons that did not exhibit any form of gain modulation (i.e., “purely visual” neurons), that is, they responded to visual stimuli at a given spatial location regardless of eye- or effector- positions. However, the majority of neurons developed gain fields, which in some cases were modulated exclusively by either eye or effector position (see, for example, top panels of Figure 4), while in other cases were modulated by both eye and effector position, resulting in multiple gain fields (bottom panels of Figure 4).

### Unsupervised learning without coordinate transformation

**Figure 5:**
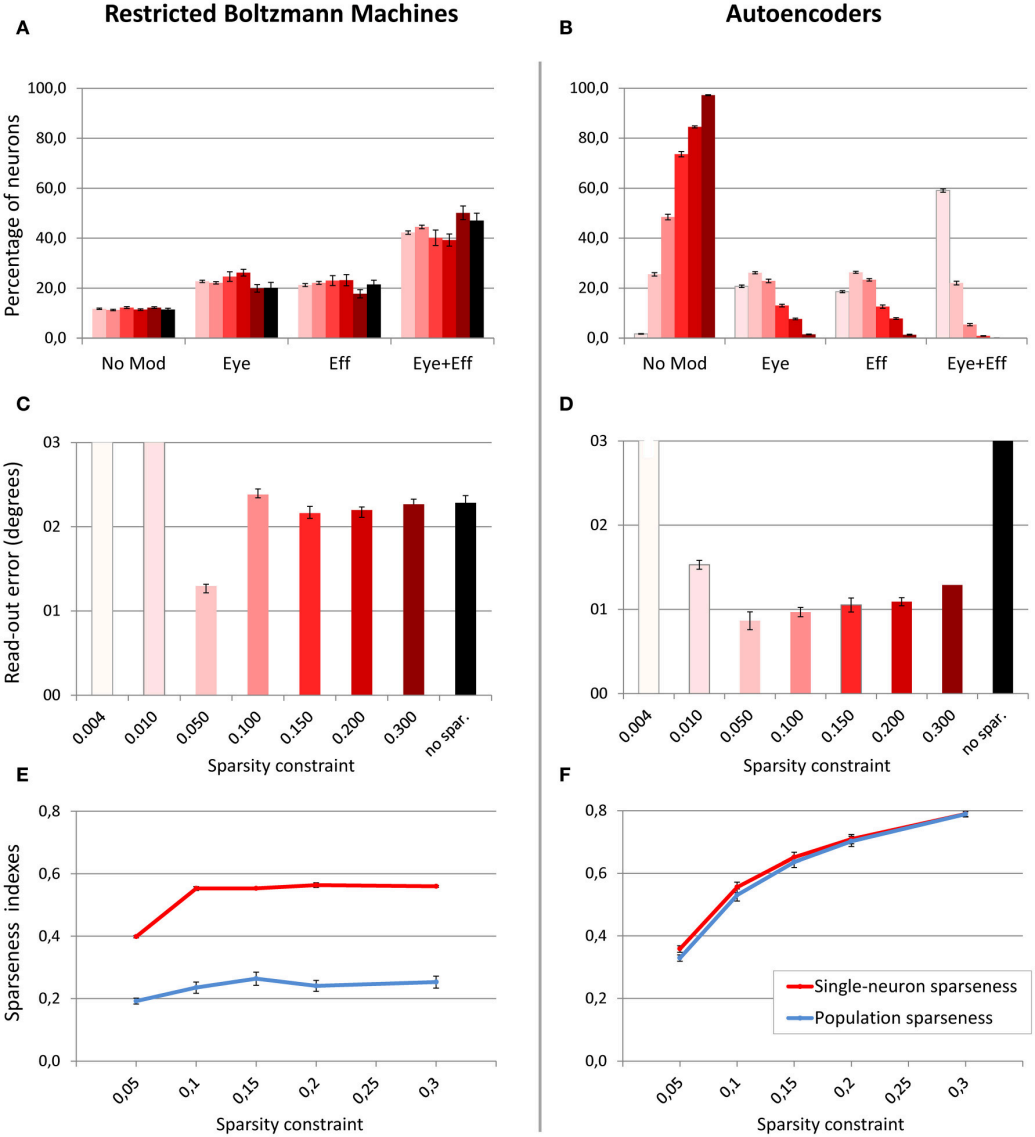
**Effect of sparsity constraints**. Distribution of gain field types emerging in the hidden neurons of RBMs **(A)** and autoencoders **(B)** with varying levels of sparsity constraint. Sparsity constraints are represented in different columns using a color scale, where lighter tones indicate stronger sparsity constraints and dark red indicates mild sparsity constraints. Read-out errors obtained at each level of sparsity constraint for RBMs **(C)** and autoencoders **(D)**. Single-neuron and population sparseness as a function of sparsity constraints for RBMs **(E)**, and autoencoders **(F)**. Note that small values indicate stronger sparseness.

In a first set of simulations, the number of hidden units was fixed to 400^4^, while the sparsity constraint was varied between 0.004 (very strong sparsity constraint, requiring low average activation) and 0.3 (mild sparsity constraint). As shown in Figure 5, the effect of sparsity constraints on the two unsupervised architectures was markedly different. Levels of sparsity constraints in the first two rows are represented using a color scale, where lighter tones indicate stronger sparsity and dark tones indicate mild sparsity. Gain modulation in RBMs (Figure 5A) was not affected by imposing sparsity constraints. In all cases, we found a modest percentage (around 10%) of purely visual neurons, which were not modulated by any postural information. A more consistent percentage of neurons (20–25%) were modulated either by eye or by effector positions, while the remaining neurons (40–50%) exhibited multiple gain fields. Read-out accuracy (Figure 5C) was always good, except for the networks trained with very strong sparsity constraints (0.01 and 0.004), where learning failed and read-out accuracy did not achieve a mean error lower than 3°. The lowest read-out error (around 1.3°) was obtained with a sparsity constraint of 0.05. In contrast, autoencoders were extremely sensitive to sparsity constraints: Strong sparsity constraints resulted in a compressed code where the majority of hidden neurons (60%) exhibited multiple gain fields (Figure 5B). When the sparsity pressure was reduced gain fields gradually disappeared, and the majority of neurons did not exhibit any modulation at all. Read-out error was generally lower compared to RBMs, and learning failed only for the networks trained with extreme (0.004) or without any sparsity constraints (Figure 5D). Notably, also for autoencoders the lowest read-out error (around 0.9°) was obtained with a sparsity constraint of 0.05, which also resulted in a distribution of gain fields more similar to that of RBMs.

Interestingly, the objective indexes of sparseness revealed that RBMs are naturally much sparser than autoencoders (see bottom panels of Figure 5). Indeed, the level of sparsity constraint turned out to have a very weak effect on population sparseness in RBMs (Figure 5E), as also confirmed by linear regression [*r*^2^ = 0.32, b = 0.05, *p* < 0.001, *n* = 50]. Single-neuron sparseness was only affected when the sparsity constraint operated below a critical level of 0.1. In order to measure what would be the “spontaneous” index of sparseness in RBMs, we trained an additional set of networks without imposing any sparsity constraint, which resulted in a single-neuron sparseness of 0.56 and a population sparseness of 0.28, showing that RBMs naturally exhibit a remarkable sparseness. In contrast, sparsity constraints in autoencoders had a marked effect on both single-neuron sparseness and population sparseness (Figure 5F), suggesting that this architecture naturally develops extremely distributed internal representations. In particular, the effect of level of sparsity constraint on population sparseness for autoencoders [linear regression: *r*^2^ = 0.88, b = 0.43, *p* < 0.001, *n* = 50] was almost one order of magnitude higher compared to RBMs. In order to measure the spontaneous index of sparseness in autoencoders, we trained an additional set of networks with a very low sparsity constraint (0.8), which is the borderline condition that still guaranteed successful learning. The latter simulations yielded sparseness values indicating non-sparse, highly distributed representations (single-neuron sparseness = 0.97; population sparseness = 0.98).

**Figure 6:**
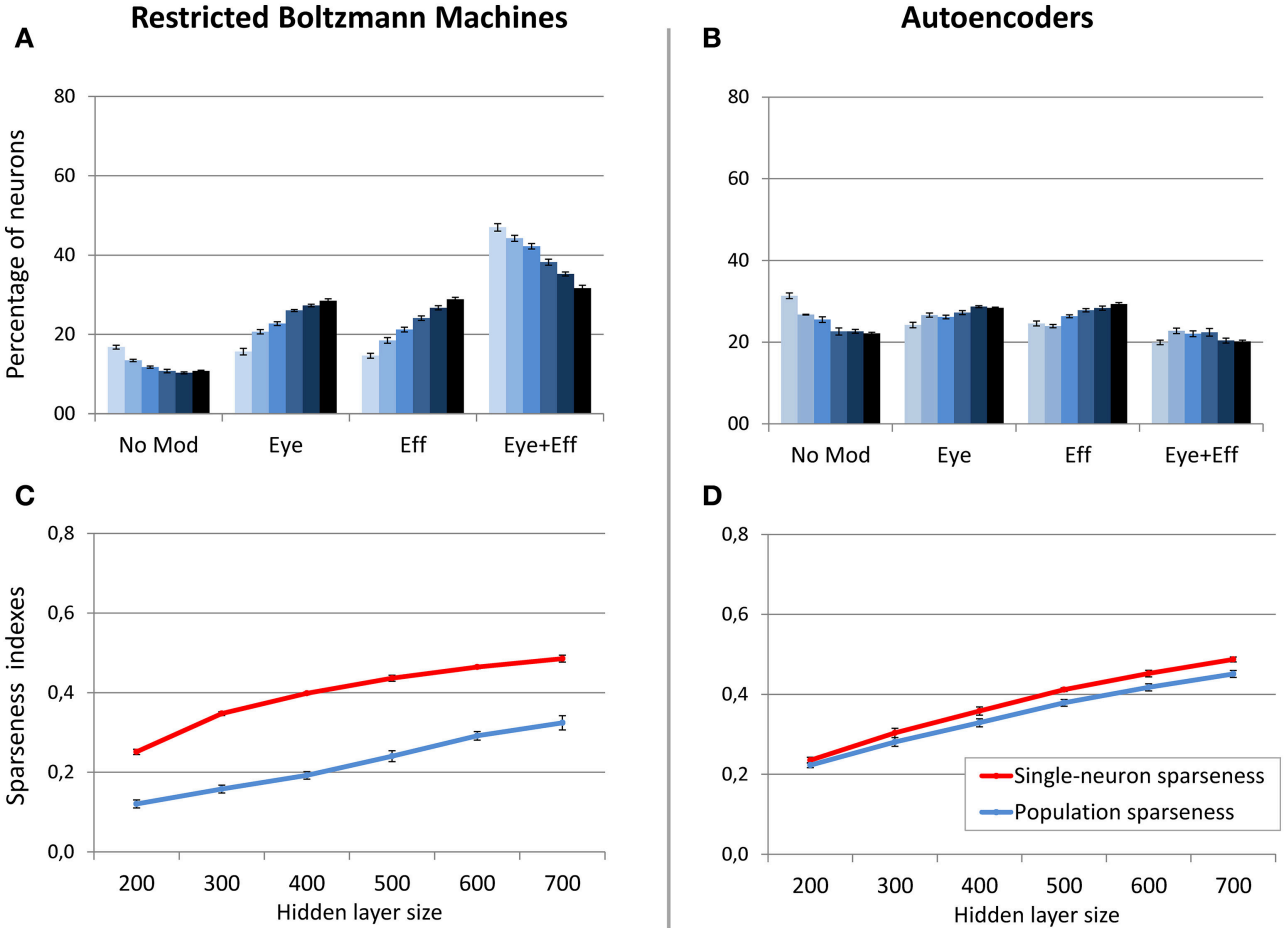
**Effect of hidden layer size, with strong sparsity constraint**. Distribution of gain field types emerging at the hidden layer of RBMs **(A)** and autoencoders **(B)** with varying number of hidden neurons and sparsity constraint fixed to 0.05. Lighter tones indicate smaller layers and dark blue indicates larger layers. Single-neuron and population sparseness as a function of hidden layer size for RBMs **(C)** and autoencoders **(D)**. Note that small values indicate stronger sparseness.

In a second set of simulations, the sparsity constraint for both architectures was fixed to the value leading to the best performance (0.05), while the size of the hidden layer was varied systematically between 200 and 700 neurons in steps of 100. This range allowed to explore the effect of relatively large increases and decreases of hidden layer sizes with respect to the previous simulations, without compromising the learning accuracy. For both architectures, the read-out accuracy was not affected by hidden layer size, and the mapping error was always below 2° (read-out errors for all different hidden layer sizes are reported in Table 1). However, as shown in Figure 6, also in this case the manipulation had different effects for the two architectures (lighter colors indicate smaller sizes). The type of encoding developed by RBMs (Figure 6A) was affected by hidden layer size: When the number of hidden neurons decreased the network developed more compressed codes, by increasing the percentage of multiple gain fields and reducing the percentage of neurons modulated by only eye or effector positions. Interestingly, it turned out that the manipulation of hidden layer size had a clear impact also on the underlying sparseness of the representation (Figure 6C). Indeed, both single-neuron and population sparseness decreased as a function of number of hidden neurons [linear regressions: single-neuron sparseness, *r*^2^ = 0.92, b = 0.22, *p* < 0.001, *n* = 60; population sparseness, *r*^2^ = 0.96, b = 0.21, *p* < 0.001, *n* = 60]. This result suggests that the distribution of gain fields in RBMs might in fact be modulated by the underlying sparseness of the representation. This was confirmed by the high correlation between the percentage of multiple gain-fields and the objective sparseness indexes [Pearson correlations: single-neuron sparseness: *r* = −0.85, *p* < 0.001; population sparseness, *r* = −0.92, *p* < 0.001].

On the contrary, neuronal tuning functions in autoencoders were not affected by hidden layer size, as this architecture always developed uniformly distributed types of gain fields (Figure 6B). Interestingly, as for RBMs the reduction of hidden layer size caused a decrease in both single-neuron sparseness and population sparseness [linear regressions: single-neuron sparseness, *r*^2^ = 0.98, b = 0.25, *p* < 0.001, *n* = 60; population sparseness, *r*^2^ = 0.98, b = 0.23, *p* < 0.001, *n* = 60]. However, the sparseness indexes did not correlate with the percentage of multiple gain-fields [all *p* > 0.05]. This suggests that similar changes in the underlying sparseness do not produce the same effect on the gain field distribution in RBMs and autoencoders.

**Figure 7:**
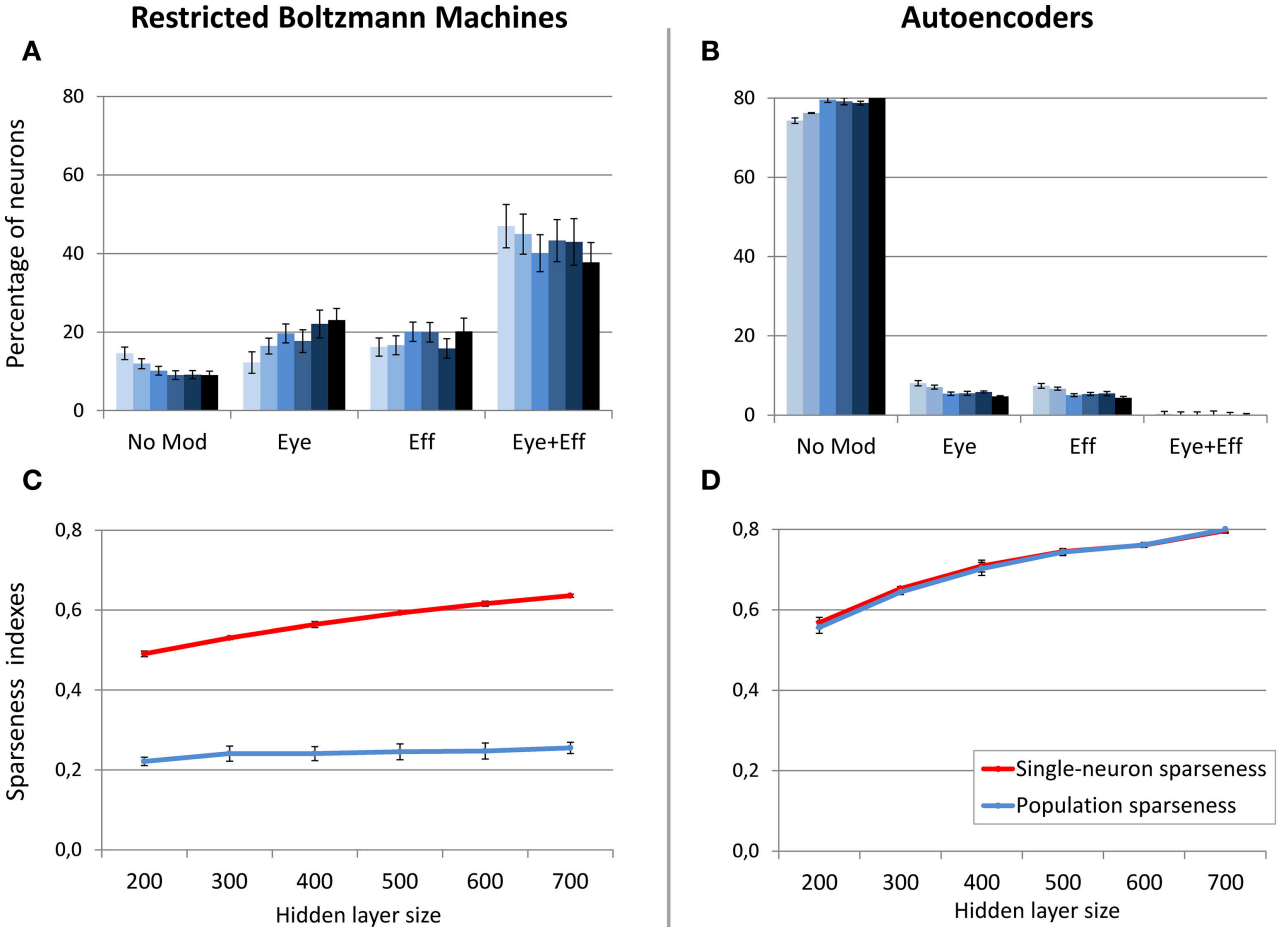
**Effect of hidden layer size, with moderate sparsity constraint**. Distribution of gain field types emerging at the hidden layer of RBMs **(A)** and autoencoders **(B)** with varying number of hidden neurons and sparsity constraint fixed to 0.2. Lighter tones indicate smaller layers and dark blue indicates larger layers. Single-neuron and population sparseness as a function of hidden layer size for RBMs **(C)** and autoencoders **(D)**. Note that small values indicate stronger sparseness.

In order to better clarify if the size of the hidden layer in RBMs modulates the distribution of gain fields only when sparseness is externally forced (i.e., when using a sparsity constraint of 0.05), in a subsequent set of simulations the sparsity constraint was set to a weak level (0.2) and the size of the hidden layer was manipulated as in the previous condition. In this case the distribution of gain fields did not systematically change (Figure 7A) but, notably, also the population sparseness was not affected (Figure 7C) [linear regression: *r*^2^ = 0.24, b = 0.03, *p* < 0.001, *n* = 60]. Correlation analyses still revealed a correlation between population sparseness and the percentage of multimodal gain fields [*r* = −0.54, *p* < 0.001], while the correlation with single-neuron sparseness was not significant [*p* > 0.05]. These results show that, for RBMs, population sparseness is a robust predictor of the distribution of gain fields: if RBMs must rely only of few active neurons to represent each sensory stimulus, they will develop more compressed spatial codes, such as those based on multiple gain fields. The corresponding simulation with autoencoders was relatively uninformative, because the weak level of sparsity constraint resulted in the absence of multimodal gain fields (Figure 7B).

### Unsupervised learning with coordinate transformation

**Figure 8:**
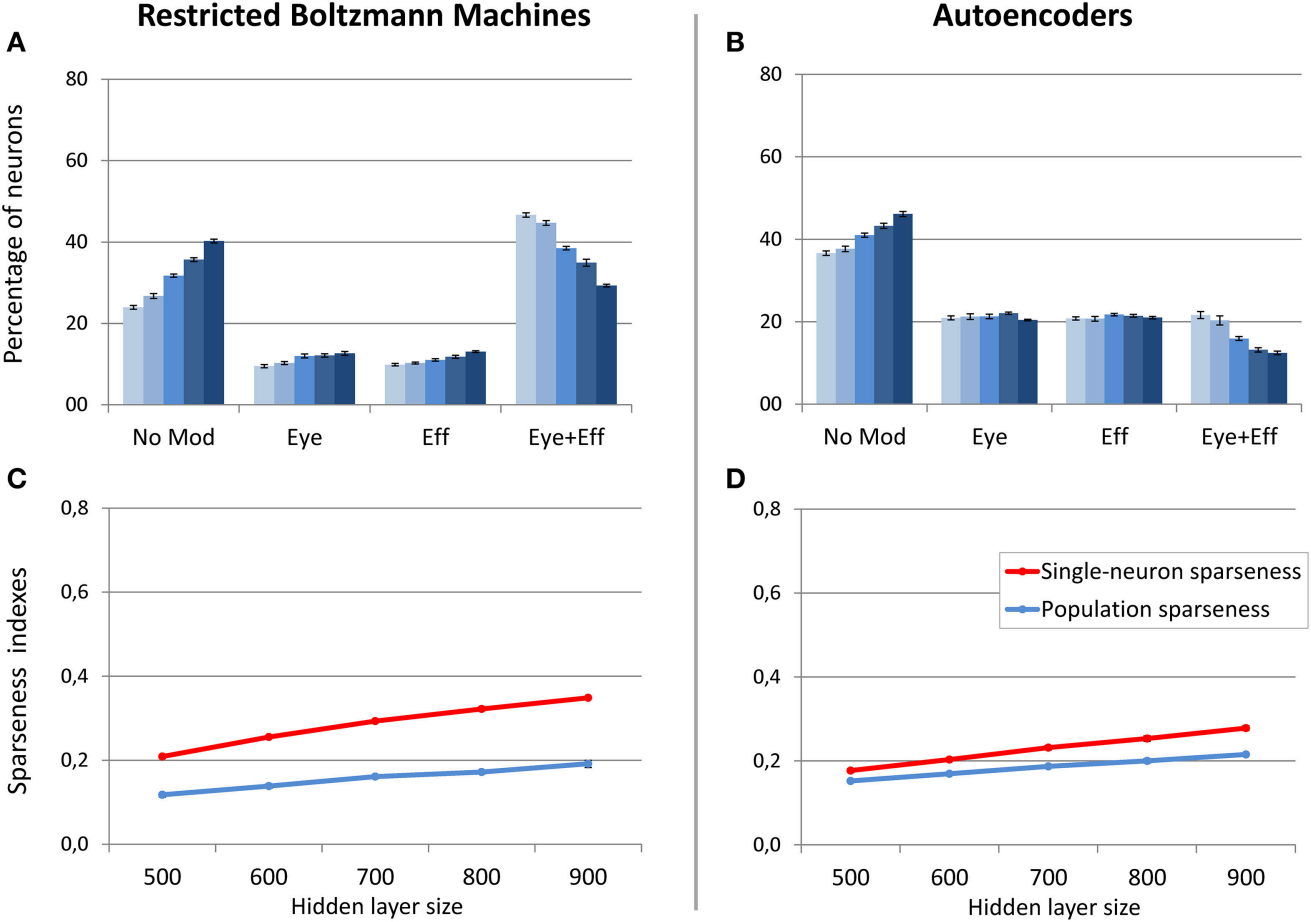
**Unsupervised learning involving coordinate transformations**. Distribution of gain field types emerging at the hidden layer of RBMs **(A)** and autoencoders **(B)** with varying number of hidden neurons. Lighter tones indicate smaller layers and dark blue indicates larger layers. Single-neuron and population sparseness as a function of hidden layer size for RBMs **(C)** and autoencoders **(D)**. Note that small values indicate stronger sparseness.

As discussed before, in this learning setting the motor program was included as input during unsupervised learning. This implies that two different coordinate systems (i.e., retinotopic and motor) are implicitly associated during training. For these simulations, we focused on hidden layer size, which was varied between 500 and 900 neurons in steps of 100. Note that the larger number of hidden neurons with respect to the previous simulations is motivated by the increased size and complexity of the training patterns. The sparsity constraint was fixed to 0.05, which was the value resulting in more accurate read-outs and more balanced distribution of gain fields for both RBMs and autoencoders in the previous set of simulations. For both architectures, read-out accuracy was always good (mapping error below 2°) and it was not affected by hidden layer size (see Table 1). As shown in Figure 8, RBMs generally developed a larger percentage of gain fields compared to autoencoders. In particular, the number of multiple gain fields was much higher for RBMs. Interestingly, for both architectures also in this case the manipulation of hidden layer size produced a systematic change in the sparseness indexes [linear regressions: RBMs single-neuron sparseness, *r*^2^ = 0.98, b = 0.14, *p* < 0.001, *n* = 50; RBMs population sparseness, *r*^2^ = 0.95, b = 0.07, *p* < 0.001, *n* = 50; autoencoders single-neuron sparseness, *r*^2^ = 0.98, b = 0.10, *p* < 0.001, *n* = 60; autoencoders population sparseness, *r*^2^ = 0.98, b = 0.06, *p* < 0.001, *n* = 60]. For both architectures, population and single-neuron sparseness were highly correlated with the percentage of multiple gain fields [Pearson correlations: RBMs single-neuron sparseness, *r* = −0.94, *p* < 0.001; RBMs population sparseness, *r* = −0.96, *p* < 0.001; autoencoders single-neuron sparseness, *r* = −0.88, *p* < 0.001; autoencoders population sparseness, *r* = −0.88, *p* < 0.001]. This finding corroborates the hypothesis that, especially for RBMs, reducing the number of active neurons results in more compressed codes based on multiple gain fields, which might be particularly advantageous in the current scenario since learning involved coordinate transformations. In contrast, fewer neurons in autoencoders exhibited multiple gain modulation (Figure 8B), even if also in this case the percentage of multiple gain fields was proportional to the underlying level of sparseness.

### Supervised learning with coordinate transformation

**Figure 9:**
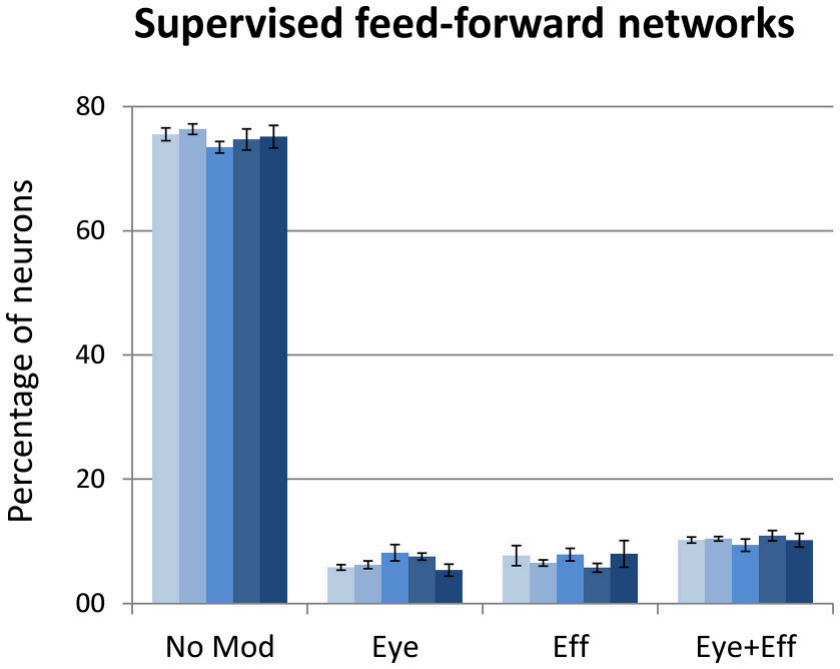
**Supervised learning of coordinate transformations**. Distribution of gain field types emerging at the hidden layer of a feed-forward, supervised neural network similar to that used by Zipser and Andersen (1988) with varying number of hidden neurons. Lighter tones indicate smaller layers and dark blue indicates larger layers.

The final set of simulations reproduced the feed-forward, supervised architecture used by Zipser and Andersen (1988). As in their original work, we did not enforce sparse coding. The size of the hidden layer was varied between 500 and 900 in steps of 100. Learning always converged and both the feed-forward mapping error and the read-out error were below 3° (see Table 1). As shown in Figure 9, this type of learning architecture developed a strikingly lower proportion of gain-modulated neurons in the hidden layer: Almost 80% of the neurons did not exhibit any form of gain field. The remaining ones were almost uniformly distributed across the three other types (about 8% for either eye or effector position; 10% for multiple gain modulation). Moreover, differently from the unsupervised architectures, the type of gain modulation was not affected by changes in the hidden layer size. This result is remarkable, because it suggests that feed-forward, supervised architectures are much less prone to develop efficient forms of space coding based on gain fields. One possible explanation for this finding is that the type of coding used to represent the motor program might have affected the efficiency of error backpropagation, which was not able to properly propagate the error signals across the hidden layer. Indeed, also Zipser and Andersen (1988) found some discrepancy between the type of gain modulations developed when using a monotonic output format compared to the Gaussian output format (which was adopted in the present study). However, the previous simulations with autoencoders showed that backpropagation can give rise to a variety of strong gain modulations when it is applied within an unsupervised learning setting. Another, more critical factor might instead be the absence of sparsity constraints, which were not used in the feed-forward models but turned out to be fundamental with autoencoders.

## Discussion

In this study we investigated the role of architectural and learning constraints in neural network models that learned to encode spatial information resulting from the combination of visual and postural signals. Results showed that, compared to the supervised architecture originally proposed by Zipser and Andersen (1988), unsupervised architectures like Restricted Boltzmann Machines (RBMs) and autoencoders discover space codes that more closely reproduce the distribution of neuronal tuning functions observed in neurophysiological experiments. In particular, the majority of hidden neurons of RBMs and autoencoders exhibited gain modulation, which in some cases only depended either on eye or effector position, while in other cases depended on both eye and effector positions, thereby resulting in multiple gain fields. In fact, all unsupervised models developed a much higher percentage of gain modulated neurons compared to the supervised models. Although the precise distribution of gain field types in the cerebral cortex depends on the exact recording site (Colby and Goldberg, 1999), our simulations suggest that this efficient form of encoding emerges more naturally if the task requires to reconstruct the whole sensory input, rather than to simply discover a feed-forward mapping to a target motor program. In other words, gain field coding might be useful when the goal is to discover “good” internal representations of the input data, that is, when the aim is to unveil and more explicitly encode the latent factors underlying the input data distribution.

As a general principle, the quality of an internal representation should reflect how well the learned features disentangle as many factors of variation as possible, at the same time discarding as little information about the data as is practical (Bengio et al., 2013). In the specific case of sensorimotor transformations, it has been proposed that good internal representations should have a variety of properties, such as the ability to combine the input signal in a nonlinear way, the ability to fully cover the range of possible input values, and the ability to represent multiple reference frames simultaneously within the same neurons (Pouget and Snyder, 2000). Populations of gain modulated neurons satisfy these requirements, allowing to encode visual space using a flexible set of basis functions. Notably, our simulations showed that this allows to learn coordinate transformations in two separate stages, by first learning the set of basis functions in a completely unsupervised way, and then learning appropriate mappings to target motor commands by relying on explicit supervision or reinforcement signals (Pouget and Snyder, 2000).

Our analyses also highlighted several differences in the spatial codes learned by RBMs and autoencoders, despite the fact that these two unsupervised architectures are often considered similar, if not equivalent (Ranzato et al., 2007; Coates et al., 2011). Even from a simple, qualitative analysis of the visual receptive fields, it turned out that these models developed different internal representations. Subsequent analyses conducted to investigate the emergence of gain fields further revealed that the distribution of hidden neurons' tuning functions in RBMs and autoencoders was similar only for a very narrow choice of the hyper-parameters. An important finding was that RBMs spontaneously exhibited a remarkable level of sparseness, which made them insensitive to external sparsity constraints, and which encouraged the emergence of compressed forms of spatial coding based on gain modulation. The spontaneous level of sparseness in RBMs could be manipulated only within a narrow range, by imposing an extreme sparsity constraint and jointly reducing the size of the hidden layer. This forced the internal representations to rely on even fewer neurons, and produced an increase in the percentage of multiple gain fields. These findings are consistent with the intuition that reducing the computational resources forces the networks to discover more complex (and compressed) forms of encoding, such as those resulting from the combination of many sensory/postural variables into multiple gain fields. Notably, for RBMs this was the case even when the task did not involve any coordinate transformations, which implied that postural variables were orthogonal. In other words, despite the fact that eye and effector positions were varied independently across training patterns, the RBMs with fewer active neurons often combined these signals together, resulting in an increase of multiple gain fields. Nevertheless, unlike autoencoders, RBMs always dedicated some representational resources also to encode eye and effector positions independently.

Autoencoders turned out to rely on much more distributed representations compared to RBMs, and were therefore extremely sensitive to external sparsity constraints. This implies that, compared to RBMs, autoencoders have an additional hyper-parameter that must be carefully tuned. Notably, when the sparsity pressure was reduced hidden neurons in the autoencoders did not develop any form of gain modulation. Only for specific values of sparsity constraints autoencoders could reproduce the variety of gain field types observed in neurophysiological data (Brotchie et al., 1995; Graziano et al., 1997; Snyder et al., 1998; Chang et al., 2009), with a distribution compatible with that of RBMs. However, in autoencoders the underlying sparseness indexes did not seem to be systematically related to the complexity of the emergent spatial codes. Though these findings alone do not allow to adjudicate between models, they call for a more systematic investigation of these different learning architectures, possibly spanning other domains and using a more direct comparison to neurophysiological data.

A plausible explanation for the striking differences in the spontaneous level of sparseness between RBMs and autoencoders can be found when considering the different processing dynamics embedded in these two neural network models. Indeed, in autoencoders the activation of each hidden neuron is deterministic, and simply corresponds to the (possibly graded) value returned by the non-linear, logistic activation function. In RBMs, instead, the value returned by the logistic function is treated as a probability, and the final activation of each hidden neuron is obtained by performing a stochastic binarization step. This important difference likely produces more sharp neuronal activations, driving RBMs to develop more sparse representations compared to autoencoders.

From a broader perspective, we believe that stochastic neural networks such as RBMs and their extension into hierarchical generative models will have an increasingly central role in neurocomputational modeling, because they provide a unique bridge between high-level descriptions of cognition in terms of Bayesian computation and low-level, mechanistic explanations inspired by the biophysical properties of real neuronal networks (Testolin and Zorzi, 2016). For example, generative neural networks are compatible with Bayesian approaches based on probabilistic population codes (Ma et al., 2006), which have been successfully used to simulate sensorimotor transformations with basis functions (Pouget and Sejnowski, 1997; Pouget and Snyder, 2000). RBMs extend the basis function approach by explaining how learning might shape the emergent neuronal gain fields, and they could similarly be combined with attractor dynamics to simulate optimal statistical inference over multisensory spatial representations (cf. Pouget et al., 2002) and spatial remapping in attention orienting (cf. Casarotti et al., 2012).

Moreover, the fact that generative networks can simulate both evoked (feed-forward) and intrinsic (feedback) neuronal activity makes them particularly suited to investigate spontaneous brain activity, which has been recognized as a fundamental property of the brain (Raichle, 2015) but whose computational role is still largely unknown. An intriguing hypothesis suggests that intrinsic activity could help with driving the brain close to states that are probable to be valid inferences once an external input arrives, thus potentially shortening the reaction time of the system (Fiser et al., 2010). Stochastic, generative networks are consistent with this “sampling-based” framework, and also support the idea that neuronal noise could play an important role during sampling (Kirkpatrick et al., 1983), for example by keeping the system in a metastable state that facilitates flexible settling into the most appropriate configuration (Kelso, 2012; Deco et al., 2013). Notably, we are also beginning to better understand how these powerful models could be implemented with biologically more realistic architectures, such as those incorporating temporal dynamics and spike-based communication (Buesing et al., 2011; Nessler et al., 2013).

In conclusion, we hope that the recent breakthroughs in neurally-inspired machine learning will attract the interest of the neuroscience community, as these models hold great promise for improving our understanding of how learning shapes and organizes information processing in complex neuronal networks.

## Author contributions

AT, MD, and MZ equally contributed to the research design. AT implemented the simulations. AT and MD analyzed the data. AT and MZ wrote the paper. All the authors are accountable for all aspects of the work in ensuring that questions related to the accuracy or integrity of any part of the work are appropriately investigated and resolved.

### Conflict of interest statement

The authors declare that the research was conducted in the absence of any commercial or financial relationships that could be construed as a potential conflict of interest.

## Footnotes

^1^http://ccnl.psy.unipd.it/research/deeplearning

^2^MATLAB provides several improved versions of the standard backpropagation algorithm. An extended set of preliminary simulations was used to establish the best performing variant. In particular, these training functions were tested: *traingdm* (gradient descent with momentum); *traingda* (gradient descent with adaptive learning rate); *traingdx* (gradient descent with momentum and adaptive learning rate); *trainscg* (scaled conjugate gradient) and *trainrp* (resilient backpropagation). The most stable and accurate learning algorithm was resilient backpropagation (Riedmiller and Braun, 1993).

^3^It turned out that more than 95% of hidden neurons responded to the visual input, with a minimum activation value exceeding a threshold of 0.1.

^4^The initial size of the hidden layer was determined empirically based on a set of pilot simulations to guarantee reliable and relatively fast convergence of learning.
